# Ratio of apoB/LDL: a potential clinical index for vascular cognitive impairment

**DOI:** 10.1186/s12883-016-0766-1

**Published:** 2016-11-25

**Authors:** Cheng Qian, Fei Tan

**Affiliations:** Department of Neurology, Shengjing hospital of China Medical University, NO.53 Huangxiang Road, Shenyang, China

**Keywords:** Vascular dementia, Vascular cognitive impairment, Stroke, MMSE, apoprotein B, Low density lipoprotein

## Abstract

**Background:**

Vascular cognitive impairment (VCI), compared to vascular dementia (VD), has a broader definition and highlights the effect of vascular disease in dementia, and stroke seems play an important role in the development of VCI. However, not all patients with brain infarcts suffer from VCI; unique risk factors appear to cause such progression. This study aimed to find potential risk factors of vascular cognitive impairment among patients with brain infarcts.

**Methods:**

Thirty-seven dementia patients and 74 brain infarction patients were included; all had infarcts in both basilar ganglia. The frequencies of risk factors, such as age, hypertension, and hyperlipidemia, were compared between the two groups.

**Results:**

The incident rate of hyperlipidemia in the patients with dementia was 35.14%, which was significantly lower than that in the patients with infarction (59.46%, *P* = 0.015). In the dementia group, there was a positive correlation between the ratio of apoprotein B (apoB)/low density lipoprotein (LDL) and the Mini Mental State Examination (MMSE) score (*R* = 0.411, *P* = 0.011).

**Conclusion:**

Our study indicated that the ratio of apoB/LDL may be a potential clinical index for vascular cognitive impairment.

**Electronic supplementary material:**

The online version of this article (doi:10.1186/s12883-016-0766-1) contains supplementary material, which is available to authorized users.

## Background

Vascular dementia (VD) is a type of dementia that results from strokes destroying the brain areas important for memory and intelligence, comprises at least 20% of dementia cases, and is the second most common cause of dementia, after Alzheimer’s disease (AD) [1]. However, it is sometimes difficult to differentiate VD and AD. In the early 90s, the diagnostic criteria of VD were mainly based on those of AD’s, which emphasized impairment of memory and activities of daily living [2]. The criteria did not take into full consideration the effects of cerebrovascular lesions on cognitive impairment; However, several clinical-pathological studies have highlighted the role of cerebrovascular disease, not only as a primary cause of vascular dementia, but also as a modifier of the expression of dementia caused by other factors, such as AD [1, 3]. Moreover, new experiments have revealed functional and pathogenic synergy between neurons, glia, and vascular cells [4–6], providing a new framework to evaluate how alterations in cerebral blood vessels could contribute to the neuronal dysfunction underlying cognitive impairment. To highlight the vascular nature of this cognitive deficit, the term vascular cognitive impairment (VCI) was introduced to better reflect the full range of cognitive alterations resulting from vascular factors [7]. VCI has been widely accepted and is currently defined as “a syndrome with evidence of clinical stroke or subclinical vascular brain injury and cognitive impairment affecting at least one cognitive domain” [1]; vascular dementia is the most severe form of VCI.

Compared with AD, VCI is less well researched; there are many studies about potential risk factors for AD, whereas research about VCI risk factors is limited. The construct of multi-infarct dementia, by attributing cognitive impairment to multiple strokes, enables prevention of cognitive impairment by controlling risk factors for stroke [8], such as hypertension, diabetes, smoking, and hyperlipidemia. However, not all patients with brain infarcts suffer from VCI; unique risk factors appear to cause such progression. This study aims to evaluate potential risk factor for VCI in patients with brain infarcts.

## Methods

### Study samples

In the present study 111 patients were included who were the inpatients of the Neurologic Department in Shengjing Hospital of China Medical University from 2010 to 2015. All patients underwent magnetic resonance examination, and their magnetic resonance imaging showed infarcts in both basilar ganglia with or without infarcts in other brain areas. Based on the presence or absence of cognitive impairment, the 111 patients were divided into two groups: dementia group (37 patients) and infarction group (74 patients). The 37 patients in the dementia group were all underwent a Mini Mental State Examination (MMSE), and diagnosed as VCI, based on the diagnosis criteria provided by Rodríguez García [9]. Exclusion criteria: disorders severe enough to cause cognitive impairment, such as major depression, intracranial neoplasia, subdural hematoma, chronic hydrocephalus, and intracranial infection. The 74 patients in the infarction group had no cognitive impairment.

### Definition of risk factors

#### Smoking

Smoking was defined as at least one cigarette per day and more than one year smoking history. Hypertension: systolic blood pressure ≥140 mmHg and/or diastolic blood pressure ≥90 mmHg. Hyperlipidemia: elevated blood lipid, defined as LDL >3.37 mmol/L and/or TG >1.69 mmol/L. Diabetes mellitus (DM): with a history of diagnosed DM or DM diagnosed by endocrinologists of our hospital based on clinical evidence. Vitamin B12 (VitB12) deficiency: serum VitB12 < 180 pg/mL. Folic acid deficiency: serum folic acid <3.1 ng/mL. Encephalomalacia foci, encephalatrophy and leukoaraiosis: all MR imagine were checked by experienced radiologists, and the definition of Malacia foci, encephalatrophy and leukoaraiosis were based on their diagnosis.

### Laboratory measurements

All patients were required to fast overnight. Blood samples were collected in the following morning. Blood sugar, blood lipids and other biochemical indicators in the serum were measured by using an automatic, quality standardized, biochemical analyzer.

### MMSE

The Mini-Mental State Examination [10] is the most commonly used bedside test of cognition, which is dominated by language-based tests, and the examination tests memory, visuoperceptual and executive functions less thoroughly; Several studies have demonstrated its diagnostic utility for dementia in clinical practice [11, 12].

### Statistical analysis

Data are shown as mean ± SD or percentage. Welch’s *t* test was used to compare the mean values in the 2 groups; *P* < 0.05 indicated statistical significance. Logistic regression was used to find potential independent risk factors. Further, linear regression was used to describe the relationship among any potential risk factors and MMSE score, and Durbin-Waston test was used to adjust the positive result.

## Results

As shown in Table 1, the incidence rate of hyperlipidemia in the dementia group (35.14%) was significantly lower than that in the infarction group (59.46%, *P* = 0.015), and the other risk factors, such as age, sex, smoke, had no significantly difference between two groups. And the logistic regression shows that hyperlipidemia is not the independent risk factor of VCI in infarction patients (Table 2).

**Table 1:** Comparison of demographic and clinical characteristics of the two study groups

	Dementia	Infarction	*P* value
Number	37	74	
Age	70.62 ± 9.95	70.82 ± 12.04	0.93
Sex	F: 37.84%	F: 45.95%	0.421
	M: 62.16%	M: 54.05%	
Smoke (%)	16.22	27.03	0.208
Hypertension (%)	72.97	71.62	0.882
Hyperlipidemia (%)	35.14	59.46	0.015
DM (%)	27.03	25.68	0.88
VitB12 deficiency (%)	32.43	18.92	0.115
Folic acid deficiency (%)	13.51	5.41	0.143
Foci of malacia (%)	29.73	20.27	0.271
Encephalatrophy (%)	81.08	66.22	0.105
Leukoaraiosis (%)	75.68	66.22	0.312

**Table 2:** Variables in the equation

	B	S.E.	Wald	df	Sig.	Exp(B)
lipid	−0.996	0.418	5.681	1	0.017	0.369
Constant	−0.223	0.274	0.664	1	0.415	0.8

Subclasses of blood lipids are listed in Table 3; The cholesterol (CHOL), TG, LDL, and apolipoprotein A-I, apolipoprotein B (apoB) levels of the patients in the dementia group values were significantly lower than those of the patients in the infarction group.

**Table 3:** Subtypes of lipids in two groups

	Dementia	Infarction	*P* value
CHOL (mmol/l)	4.30 ± 0.86	4.82 ± 0.90	0.004
TG (mmol/l)	1.22 ± 0.51	1.55 ± 1.07	0.032
HDL (mmol/l)	1.11 ± 0.4	1.13 ± 0.25	0.770
LDL (mg/dl)	1.08 ± 0.31	1.26 ± 0.35	0.006
apoA-I (mg/dl)	1.13 ± 0.19	1.23 ± 0.27	0.039
apoB (mg/dl)	0.91 ± 0.22	1.05 ± 0.27	0.005

But logistic regression also found that the above five lipids index were not independently associated with the VCI study group (Table 4).

**Table 4:** Variables in the equation

	B	S.E.	Wald	df	Sig.	Exp(B)
TG	−0.634	0.402	2.489	1	0.115	0.530
Chol	0.511	0.825	0.384	1	0.536	1.667
LDL	−1.944	2.172	0.801	1	0.371	0.143
ApoA-I	−2.171	1.078	4.057	1	0.044	0.114
ApoB	−1.004	2.411	0.173	1	0.677	0.366
Constant	3.639	1.575	5.335	1	0.021	38.052

**Fig. 1:**
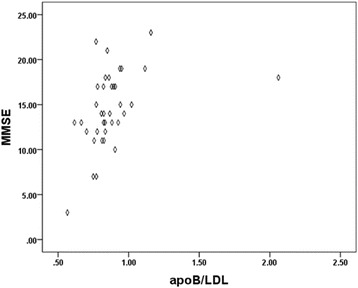
Scatter diagram of the ratio apoB/LDL ratio and MMSE score

When compared the five lipids index with MMSE score in the dementia group, there were no significantly correlation between them, however, we found that the apoB/LDL ratio was positively related to the MMSE score (*R* = 0.411, *P* = 0.011, Fig. 1, Table 5), and the linear regression demonstrated a linear relationship between the ratio of apoB/LDL and the MMSE score (*R* ^2^ = 0.169, *P* = 0.011, Table 6), but according to Durbin-Waston (DW) test [13], the DW value approaching 0 indicated positive auto-correlation, therefore this result has no statistical significance (DW = 0.451, Table 6).

**Table 5:** Correlations

		MMSE	apoB/LDL
MMSE	Pearson Correlation	1	0.411^a^
	Sig. (2-tailed)		0.011
	N	37	37
apoB/LDL	Pearson Correlation	0.411^a^	1
	Sig. (2-tailed)	0.011	
	N	37	37

^a^Correlation is significant at the 0.05 level (2-tailed)

**Table 6:** Model summary^b^

Model	R	R Square	Adjusted R square	Std. error of the estimate	Durbin-Watson
1	0.411^a^	.169	0.146	3.88304	0.451

^a^Predictors: (Constant), apoB/LDL

^b^Dependent Variable: MMSE

## Discussion

The present study showed two interesting findings: 1) Lipid levels of the dementia patients were lower than those of the infarction patients in our study; 2) The apoB/LDL ratio appeared to be positively related to the MMSE score in the dementia patients.

Hyperlipidemia is abnormally elevated levels of any or all lipids and/or lipoproteins in the blood. Based on the density, lipoproteins consist of many subtypes, including LDL, very low density lipoprotein (VLDL), intermediate low density lipoprotein (ILDL), high density lipoprotein (HDL), and chylomicron (CM). A different density determines a different fate of the particle and its influence on metabolism. Among these lipoproteins, LDL is generally considered a “bad” lipoprotein, meaning the strongest causative risk factor for atherosclerosis [14]. According to the ‘response-to-retention hypothesis’ [15], LDL is bound and retained by extracellular matrix components; undergoes a series of modifications in the arterial wall, including oxidation, lipolysis, and proteolysis; fuses together; and over time, increasing quantities of LDL aggregate in the arterial wall, triggering a cascade of inflammatory and apoptotic responses. The aforementioned progress is called atherogenesis, which leads to several cardiovascular diseases.

Furthermore, studies have suggested that cardiovascular risks contribute to VD, cognitive impairment and AD, and hyperlipidemia is one of the atherogenic factors among those cardiovascular risks [1, 16, 17]. However, not all lipoproteins and lipids are harmful, and even the “bad” lipoprotein, LDL, is essential for physiologic needs. Several studies found no relationship between hyperlipidemia and cognitive impairment [18, 19], and some studies reported potential benefits of lipoproteins and lipids. Ledesma [20] demonstrated that cholesterol loss during aging occurs in rodents’ and humans’ hippocampus, and Mauricio’s [21] study suggested that this loss would contribute to cognitive decay in the aged. Reynolds [22] suggested that in humans, higher apoB and total cholesterol levels were advantageous to cognitive health, though effects diminished by age 65. In addition, one study even demonstrated that high total cholesterol levels in late life were associated with a reduced risk of dementia [23]. Our study found that the percentage of hyperlipidemia in the dementia group was lower than that in the infarction group; although, this does not prove that hyperlipidemia is a protective factor for vascular dementia, considered together with above studies, lipids appear to have complex effects in cognitive health.

Apolipoproteins are proteins that bind lipids to form lipoproteins. They transport lipids through the lymphatic and circulatory systems. Apolipoprotein B100 (apoB100), one of the primary apolipoproteins, which is synthesized in the liver, and it is an essential apolipoprotein of VLDL, ILDL, and LDL. Because LDL persists longer in the plasma than VLDL and IDL, 90% or more of all apoB100 is associated with LDL. Therefore, many studies consider apoB the same risk factor as LDL [24–26].

However, apoB is simply a transportation molecule, and LDL is not the only passenger, especially in humans. The intestine synthesizes a truncated form of apolipoprotein B (apoB48), which is the essential apolipoprotein of CM. In addition, Neda’s [27] study demonstrated evidence of no association of apoB polymorphisms (XbaI) with obesity and serum lipid levels, indicating that the LDL and apoB may not match with each other in quantity. Furthermore, the discordance between apoB and LDL may not be a rare clinical phenomenon [28]; another study [29] showed that the superiority of apoB and LDL for cardiovascular risk assessment is most evident when LDL and apoB are discordant, and the discordance, LDL > apoB, was associated with insulin resistance, smaller LDL particle size, increased systemic inflammation and lower circulating levels of serum lipids, whereas discordance in the other direction, apoB > LDL, was associated with larger LDL particle size, elevated levels of lipoprotein a, and lipoprotein-associated phospholipase A2. Moreover, small size LDL particles may be one of multiple atherogenic triggers for the pathology of AD [30], and systemic inflammation and insulin resistance are also considered potential risk factors of dementia [31–33].

Of note, apoB itself might also have some benefits: a previous study [34] demonstrated that apoB, either isolated or in LDL, exhibits activity of both phospholipase A1 and phospholipase A2, and apoB may play a protective role by removing oxidized fatty acids from the sn-2 position of LDL phospholipids to reduce its cellular uptake. ApoB might also contribute to innate defense; studies reported that apoB100 and apoB48 could prevent Staphylococcus aureus (S. aureus) infection by binding and sequestering S. aureus and antagonizing quorum sensing, thereby reducing virulence [35, 36].

In addition, higher levels of LDL may contribute to alterations in brain tissue structure, particularly white matter integrity, leading to cognitive impairment and dementia [37]. Notably, Wang [38], using ldlr−/− mice, showed that higher levels of LDL, together with increased intracellular lipid deposition, impaired spatial cognition, decreased synapse density, and increased neuronal apoptosis in the hippocampus. In light of the above insights about apoB, the ratio of apoB/LDL appears a reasonable clinical index; higher apoB and lower LDL may benefit the cognitive health in VCI patients.

This question warrants consideration: How might serum lipoprotein and apolipoprotein contribute to the development of VCI? Normally, changes in serum cholesterol do not affect brain cholesterol homeostasis, and there seems to be no relevant cholesterol flux from the periphery into the brain [39]. Transport of serum lipids through blood-brain barrier (BBB) may be explained by the conversion of cholesterol to oxysterols [37], cholesterol may undergo an oxidative process that allows the resultant serum-derived 27-hydroxycholesterol (27-OHC) and brain-derived 24S-hydroxycholesterol (24S-OHC) to freely diffuse into the brain driven by concentration gradients [40]; 27-OHC and 24S-OHC have been considered biomarkers for certain neurodegenerative disease including AD [41]. In addition, dyslipidemia may alter BBB function in AD [42], contributing to the transportation of inflammatory lipids from the blood [6]. Further, studies have found ApoB-100 protein in the membrane microdomains of primary brain endothelial cells [43, 44]. Using the LDL-receptor binding domain of apoB could be an effective way to deliver recombinant proteins across the BBB, and such an approach can be used to treat a neurodegenerative disease [45, 46].

This study has some limitations. First, compared fewer samples, more patients should be studied. Second, the MMSE has not been adjusted for the lack of the educational experience of some patients, which this could lead to a certain error. Third, without other examinations, such as the Hachinski Ischemic Score, we could not define the subclass of dementia, so we used the broader concept: VCI. However, our sample only included a small subclass of VCI. Most patients were likely cases of multi-infarct dementia, according to the MRI findings and diagnostic criteria provided by Rodríguez García [9]; this may affect the generalizability of our results. Fourth, the age of the infarction group is relatively young compared with dementia group, although *P* = 0.93 > 0.05, with no statistical significance, but it still can’t exclude the possibility that the infarction group would develop cognitive impairment at a later life. Fifth, in our sample, the infarcts were multi-focal and some had encephalomalacia foci, but most had a scattering lacunar infarcts; consequently, it was difficult to measure the area of infarcts, so we could only compare the frequency of patients with encephalomalacia foci, encephalatrophy, and leukoaraiosis between the two groups, but still the lack of cognitive impairment in the infarction group could just be the result of insufficient lesions. Sixth, due to the *R* value is more than 0.4 but less than 0.8, the positive correlation between the ratio of apoB/LDL and MMSE score is moderated, on the one hand, the low *R* value means that the model of apoB/LDL could not fully explain the change of MMSE score, there must be other factors, and it could lead to the negative result of DW test, so our finding was not comprehensive, but from current data, we couldn’t find the other factors, more studies should be done, on the other hand, yet, it at least means that the model of apoB/LDL play a certain role in the change of MMSE score.

## Conclusion

In summary, lipid may be the key factor causing VCI in the infarction patients, hyperlipidemia was less common in VCI compared with stroke without dementia, and to our best knowledge, this article is the first ever to present the ratio of apoB/LDL as a possible clinical index for VCI. Further studies should be performed based on the new insights this study of VCI provides.

## Additional file

Additional file 1: Original data set﻿. (XLS 41 kb)

## Abbreviations

24S-OHC: 24S-hydroxycholesterol
27-OHC: 27-hydroxycholesterol
AD: Alzheimer’s disease
apoB: apoprotein B
apoB100: apolipoprotein B100
apoB48: apolipoprotein B48
BBB: Blood-brain barrier
CHOL: Cholesterol
CM: Chylomicron
DM: Diabetes mellitus
DW: Durbin-Waston
HDL: High density lipoprotein
ILDL: Intermediate low density lipoprotein
LDL: Low density lipoprotein
MMSE: Mini mental state examination
S. Aureus: Staphylococcus aureus
VCI: Vascular cognitive impairment
VD: Vascular dementia
VitB12: Vitamin B12
VLDL: Very low density lipoprotein
